# Implantable cardioverter-defibrillator in patients awaiting heart transplantation: Reinforcing the bridge to transplant

**DOI:** 10.1016/j.hroo.2021.11.003

**Published:** 2021-12-17

**Authors:** Jonathan P. Ariyaratnam, Michael B. Stokes, Dennis H. Lau

**Affiliations:** Department of Cardiology, Royal Adelaide Hospital and Centre for Heart Rhythm Disorders, The University of Adelaide, Adelaide, Australia

Heart transplantation remains the only curative therapy for end-stage heart failure, offering excellent 5-year survival rates of up to 72% for patients with otherwise extremely bleak prognostic outlooks.^1^ However, a significant challenge for heart transplant candidates is surviving the pretransplant waitlist time, which can be considerable owing to donor organ availability. Reassuringly, waitlist survival rates have improved dramatically in recent years, with 1-year survival on the waitlist increasing from 34.1% in 1987–1990 to 67.8% in 2011–2017.^2^ Factors influencing these improved waitlist survival rates include improvements in patient selection, development and utility of prognostic pharmacological therapies in heart failure, and increased implementation of mechanical circulatory support such as left ventricular assist devices (LVAD) as a bridge to cardiac transplantation. In this issue of *Heart Rhythm O*^*2*^, Lin and colleagues^3^ evaluated the potential value of implantable cardioverter-defibrillator (ICD) therapy in improving waitlist survival rates in those with end-stage heart failure who may not fulfil the standard guideline criteria for ICD implantation.

This analysis identified 10 studies comparing outcomes in 36,112 heart transplant candidates with and without ICDs between 1992 and 2014, thereby covering a period over which heart failure management (both pharmacological and nonpharmacological) has evolved considerably. Despite the cohort in this meta-analysis representing a symptomatic advanced heart failure population, only 62.5% of these patients had an ICD. The outcomes assessed included total mortality, sudden cardiac death, non–sudden cardiac death, and survival to heart transplantation. Using a random-effects model for calculating unadjusted pooled risk ratios, the authors showed that ICDs conferred a statistically significant 40% reduction in total mortality, 73% reduction in sudden cardiac death, and 9% increased chance of surviving to heart transplantation. An adjusted analysis confirmed a significant reduction in total mortality with ICDs. On the other hand, non–sudden cardiac death was not significantly reduced by the presence of an ICD. Further subanalyses showed that there was no difference in total mortality when comparing primary prevention ICDs with secondary prevention ICDs, while the proportion of patients receiving inappropriate ICD therapies was low, at 5%–7%, and those receiving appropriate ICD therapies was 26% (15%–65%).

The authors of this study are to be commended for highlighting an important issue with respect to the management of patients with end-stage heart failure listed for heart transplantation. The finding that ICDs were associated with reduced all-cause mortality rates and increased heart transplantation rates, driven largely by a dramatic reduction in sudden cardiac death, suggests an ongoing incidence of life-threatening ventricular arrhythmias in this cohort of patients. Current guidelines do not recommend ICDs for most NYHA class IV heart failure patients, as the risks of cardiac decompensation and pump failure outweigh those of sudden cardiac death.^4^^,^^5^ However, heart transplant candidates represent a unique cohort of NYHA class IV heart failure patients in whom every effort is made to reduce the risk of pump failure through use of inotropic support and mechanical circulatory devices. With this reduced impact of pump failure, the risk of sudden cardiac death assumes greater relevance, and Lin and colleagues highlighted this by reporting that the number of patients reaching transplant can be improved through negating the effect of sudden cardiac death. Furthermore, many new-generation ICDs now exhibit novel algorithms for the remote detection of pulmonary congestion indicative of impending cardiac decompensation to facilitate early identification of cardiac decompensation and intervention to reduce hospitalization.^6^ Such technological advancement may add additional benefits of ICDs in heart transplant candidates beyond sudden cardiac death reduction.

Although the paper presents compelling data, several limitations should be considered. The populations studied are likely not representative of a contemporary heart transplant population, as the majority of studies included patients enrolled more than 10 years ago. Patients listed for heart transplant in the current era are likely to be older and have more severe heart failure.^5^ Additionally, heart transplant candidates today are significantly more likely to be bridged to transplant with mechanical circulatory support. While the proportion of patients with LVADs in this study was 27%, the overall prevalence of LVADs in patients listed for heart transplant in 2018 in the United States was 43.6%.^7^ Notably, overall survival to transplant for patients with LVADs has increased, from 10% in 1996–2000 to 70% in 2011–2017.^2^ One of the most important developments in LVAD technology is the change from pulsatile to continuous-flow LVADs, which have been shown to be associated with better survival and are now more commonly used.^8^ While recent evidence suggests that ICD use is associated with reduced mortality in patients with LVADs, this benefit has not extended to studies of patients with continuous-flow LVADs.^9^ Therefore, whether the results of this study can be extended to contemporary heart transplant candidates remains unclear. Additionally, the results of this analysis may be confounded by selection bias, as most of the included patients (99%) had ICDs implanted prior to heart transplant listing, leaving minimal evidence to support the implantation of ICDs in patients already listed for heart transplant.

Notwithstanding the findings of this study, several additional challenges may continue to limit the use of ICDs in heart transplant candidates. First, primary prevention ICDs are recommended for patients with heart failure and no improvement in ejection fraction after at least 3 months of optimal medical therapy.^4^^,^^5^ The feasibility of such a delay in ICD implant may be limited in a select group of patients listed for heart transplant who develop severe acute heart failure. Yet, implantation of ICD in these patients would sit outside of current clinical guidelines and remain unsupported by clinical evidence. Second, the choice of ICD types require careful consideration given reports of significant electromagnetic interactions between subcutaneous ICDs and LVADs, including increased risk of undersensing and inappropriate shocks.^10^ Last, there is increasing recognition of the issue of retained fragments of ICD leads following transplant, with a single-center cohort study reporting a not insignificant incidence of 27%, implicating future risk of upper extremity deep vein thrombosis and preventing the use of magnetic resonance imaging.^11^

This study serves as an important reminder to both heart failure and electrophysiology physicians regarding the clinical significance of sudden cardiac death and survival in those who are candidates for heart transplant. A prospective evaluation of this issue will remain a challenge owing to the ethics of withholding ICD implantation in a sick population with poor reserve to survive a sudden cardiac death event. Taken together, while this meta-analysis of ICDs in heart transplant candidates purports a significant role of ICDs in reinforcing the bridge to transplant by increasing survival to cardiac transplantation and reducing sudden cardiac death, clinical equipoise, multidisciplinary input, and shared decision-making remain essential when considering ICD implantation for this select cohort of heart failure patients.

## Funding Sources

Dr Ariyaratnam is supported by a postgraduate scholarship from the 10.13039/501100001786University of Adelaide. Dr Lau is supported by a Mid-Career Fellowship from 10.13039/100009727The Hospital Research Foundation.

## Disclosures

Dr Lau reports that the 10.13039/501100001786University of Adelaide has received on his behalf lecture and/or consulting fees from Abbott Medical, Boehringer Ingelheim, Biotronik, Medtronic, MicroPort CRM, and Pfizer.

## Authorship

All authors attest they meet the current ICMJE criteria for authorship.
